# Hidradenitis Suppurativa at an Uncommon Site: A Review of Its Clinical Features, Diagnostic Difficulties, and Management

**DOI:** 10.7759/cureus.18704

**Published:** 2021-10-12

**Authors:** Leo M Harvey, James K Fortson

**Affiliations:** 1 Otolaryngology, ENT Associates of South Atlanta, Atlanta, USA

**Keywords:** hidradenitis suppurativa, acne inversa, abscess, fistula, sinus tracts and tunnels

## Abstract

Hidradenitis suppurativa (HS) is a non-contagious chronic inflammatory and often debilitating skin disease that is characterized by recurrent painful nodules, draining sinus tracts, and abscesses. The disease primarily affects the axillary, perineal, inguinal, intermammary, and inframammary regions with an estimated global prevalence rate of up to 4%. The etiology of HS is still unknown, but our understanding of its pathogenic process has evolved. Once thought to be an infectious process of the apocrine gland, HS is now considered a disease of follicular occlusion.

This study aimed to discuss hidradenitis suppurativa in an uncommon site and review the clinical features, diagnostic difficulties, and management of the condition. A PubMed literature search for case reports was done using the medical subject heading (MeSH) term hidradenitis suppurative. Only reports in the last five years that were published in English were considered. The patient underwent a surgical incision and drainage of the deep neck abscesses. The patient continued to be monitored by ENT and was compared to other cases reported in this study.

HS mostly presents in the axillary, perineal, inguinal, and gluteal regions. This is a case report of HS in the neck region which is a rare location. After surgical intervention, the patient required prolonged antibiotic therapy for the resolution of symptoms.

The diagnosis of HS is made clinically and is based on typical lesions, location, and chronicity. However, phenotypic variation makes diagnosis and severity assessment difficult. Furthermore, a diagnostic delay is evident partly due to early lesions of HS mimicking other skin conditions. CT scans and ultrasounds are emerging as important diagnostic tools, especially in the case of deep-seated lesions. Multiple comorbidities are associated with HS and persistent hidradenitis suppurativa often results in complications. The recurrent nature of HS as well as the lack of curative therapies makes the treatment of the disease challenging.

## Introduction

Hidradenitis suppurativa (HS), also known as acne inversa, is a chronic inflammatory skin disease characterized by recurrent painful nodules, draining sinus tracts, and abscesses [1]. The disease primarily affects the axillary, perineal, inguinal, intermammary, and inframammary regions [2]. HS has an estimated global prevalence of up to 4.1% and an estimated 0.7-1.2% in the European-United States population [1]. Historically, HS was thought to be an infectious process of the apocrine gland but is now considered a disease of follicular occlusion [2]. The etiology of HS is still uncertain; however, three pathogenic processes are indicated; follicular hyperkeratosis with dilation, a follicular rupture that triggers a subsequent inflammatory response, and chronic inflammation with architectural changes [1].

The disease usually presents clinically with tender subcutaneous nodules that may then rupture, forming painful deep dermal abscesses and eventual fibrosis and formation of extensive sinus tracts. HS is divided into three stages based on severity called Hurley stages. Hurley stage I is characterized by a single nodule with no sinus tracts, stage II has more than one nodule with little tunneling, and stage III has multiple nodules with a lot of sinus tracts and scars involving an entire area of the body [3]. 

Multiple comorbidities are associated with hidradenitis suppurativa, and persistent HS often results in complications. The most common comorbidities include obesity, metabolic syndrome, inflammatory bowel disease, and spondyloarthropathy. Notable complications include infection, scarring with associated restricted limb mobility, obstructed lymph drainage, fistulas, and social isolation.

## Case presentation

**Figure 1:**
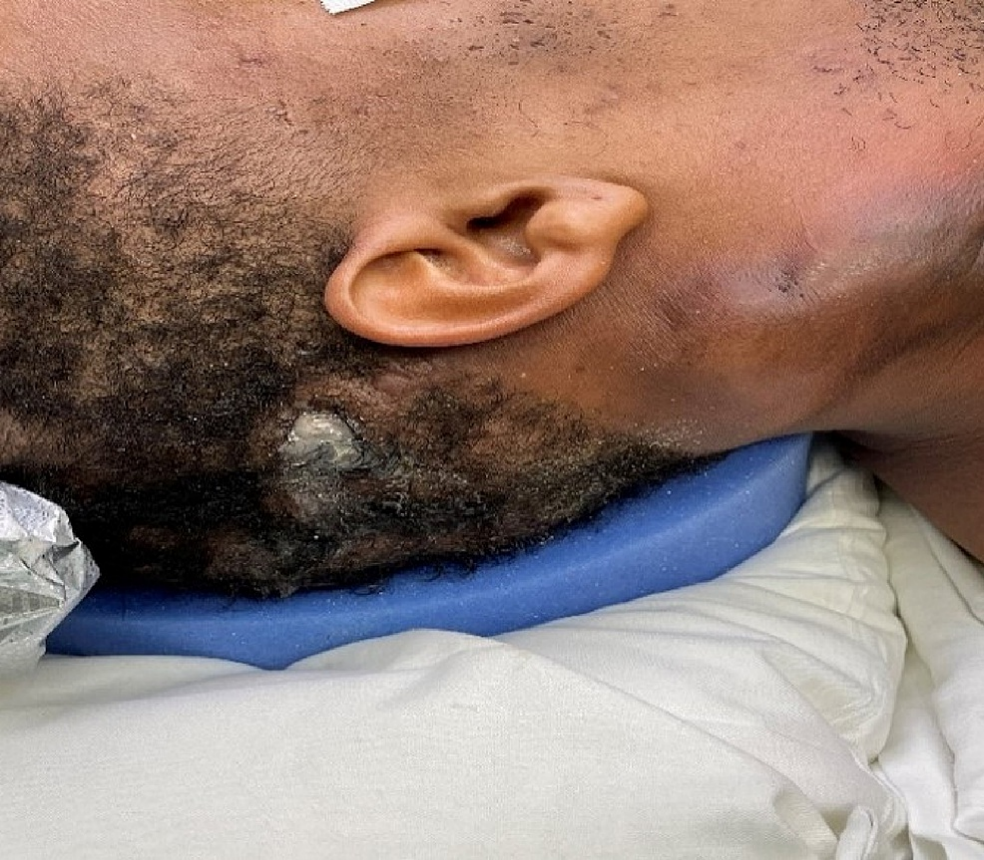
Neck mass extending from right mastoid to anterior neck with scalp drainage.

**Figure 2:**
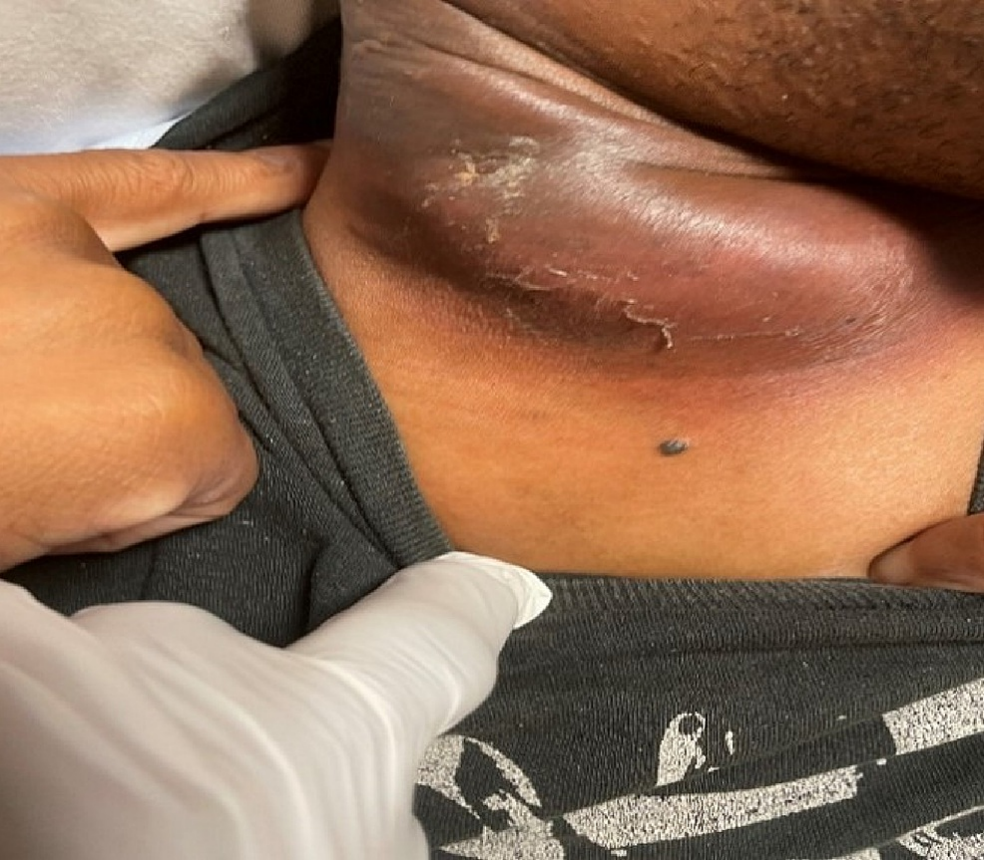
Fluid-filled mass of right anterior neck.

A 31-year-old African American male with a five-year history of hidradenitis suppurativa presented to the emergency department for an abscess of the right neck. The patient complained of dysphagia, food intolerance, and headaches. The patient was febrile and stated that the abscess is draining and painful. His medical history was negative for illicit drug use, sexually transmitted disease, or trauma. His blood pressure was 120/65 mmHg, respiratory rate was 17 breaths/min, heart rate was 116 beats/min, the temperature was 100.5°F, and oxygen saturation was 98% on room air. Physical examination showed a large neck abscess extending from the right mastoid to the anterior neck with tenderness and some scalp drainage (Figures 1, 2).

**Figure 3:**
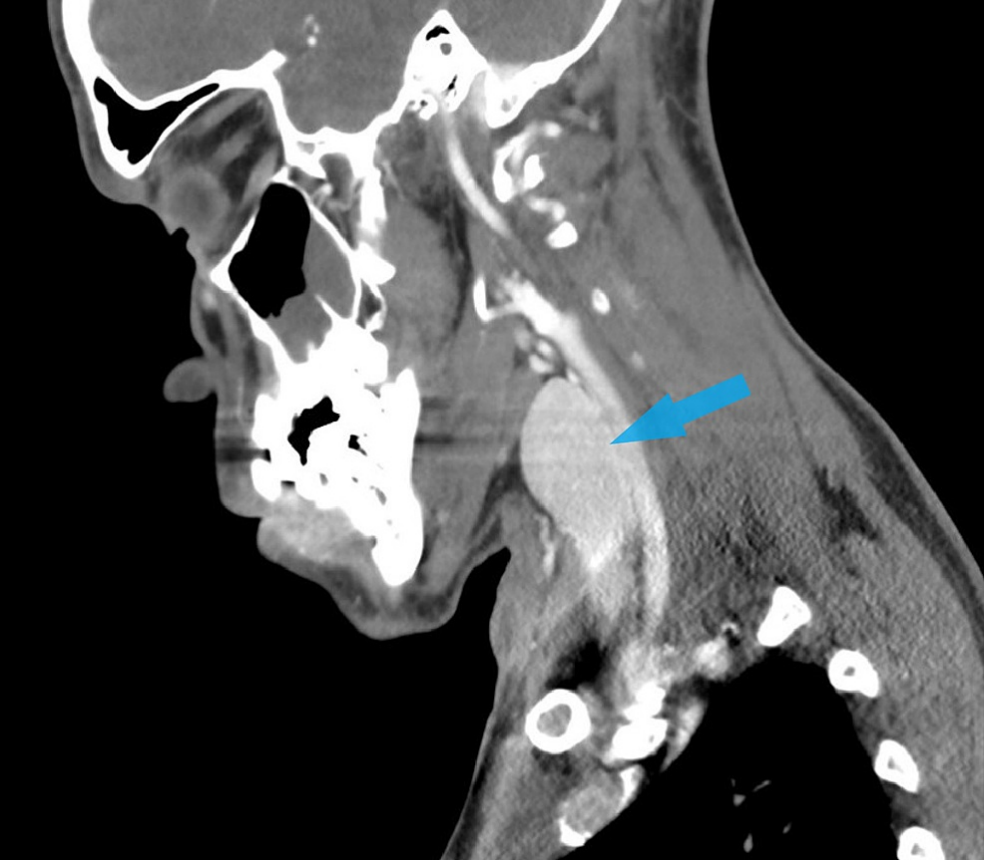
CT soft tissue neck with IV contrast showing HS abscess in the right neck. HS: hidradenitis suppurativa

**Figure 4:**
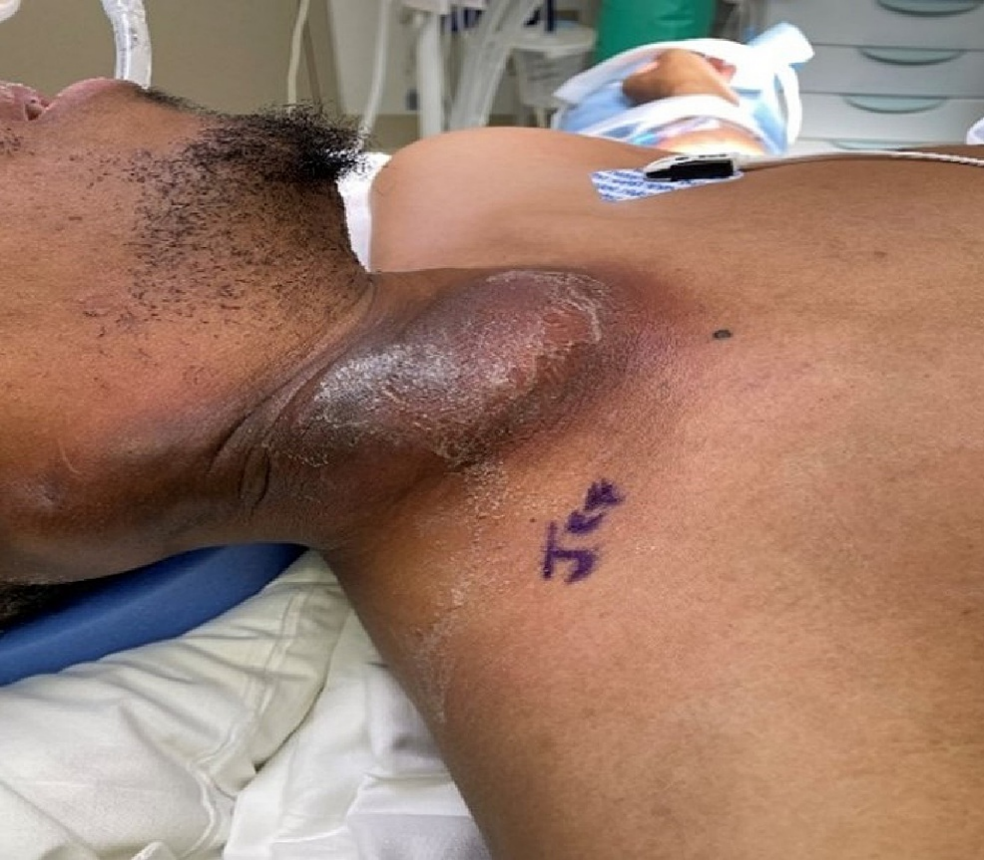
Right neck mass with fluid collection prior to I&D. I&D: incision and drainage

A CT scan of the neck with IV contrast revealed fluid collection with a thick, peripherally enhancing rim in the soft tissues of the right posterior neck (Figure 3). The abscess extended from the superficial soft tissues of the right occipital region and traveled inferiorly and anteriorly to the approximate level of the thyroid. Soft tissue edema was noted up to the level of the sternoclavicular joint (Figure 4). Nodular thickening was also observed in the right and left posterior scalp soft tissues along with persistent cervical lymphadenopathy. An incidental finding of mucosal thickening in the maxillary sinus was noted. The patient was started on vancomycin 1500 mg in 500 mL IV.

**Figure 5:**
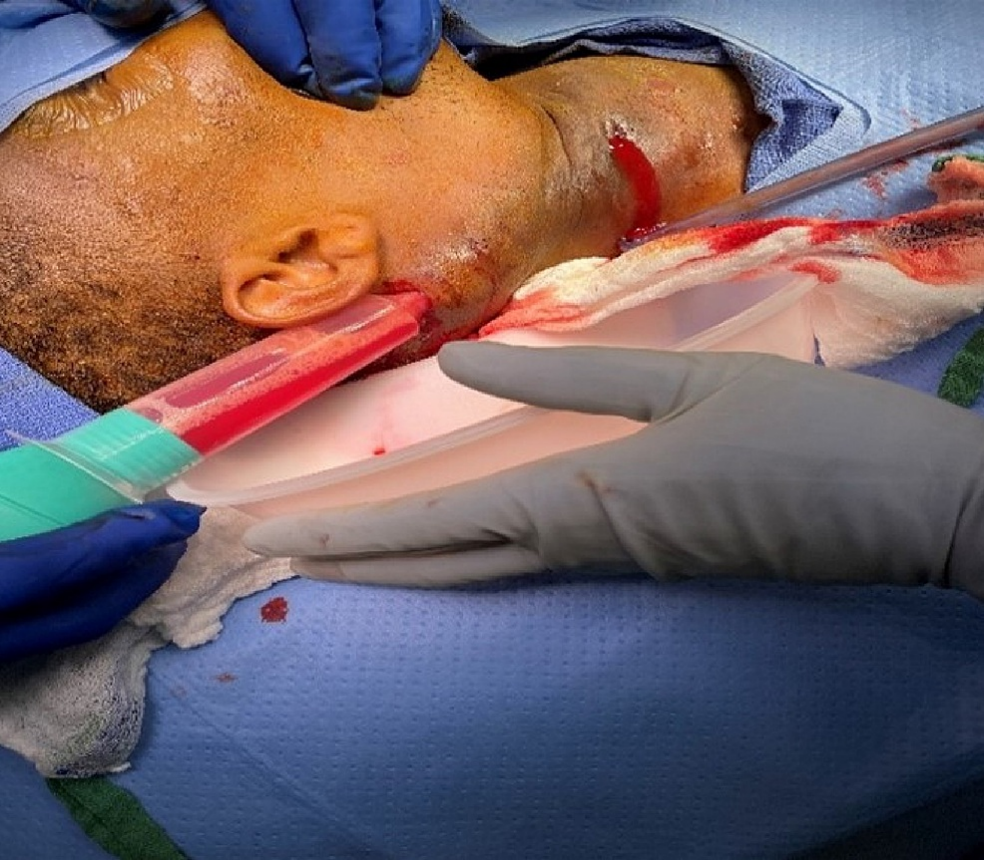
Incision, drainage, and irrigation of right neck mass.

**Figure 6:**
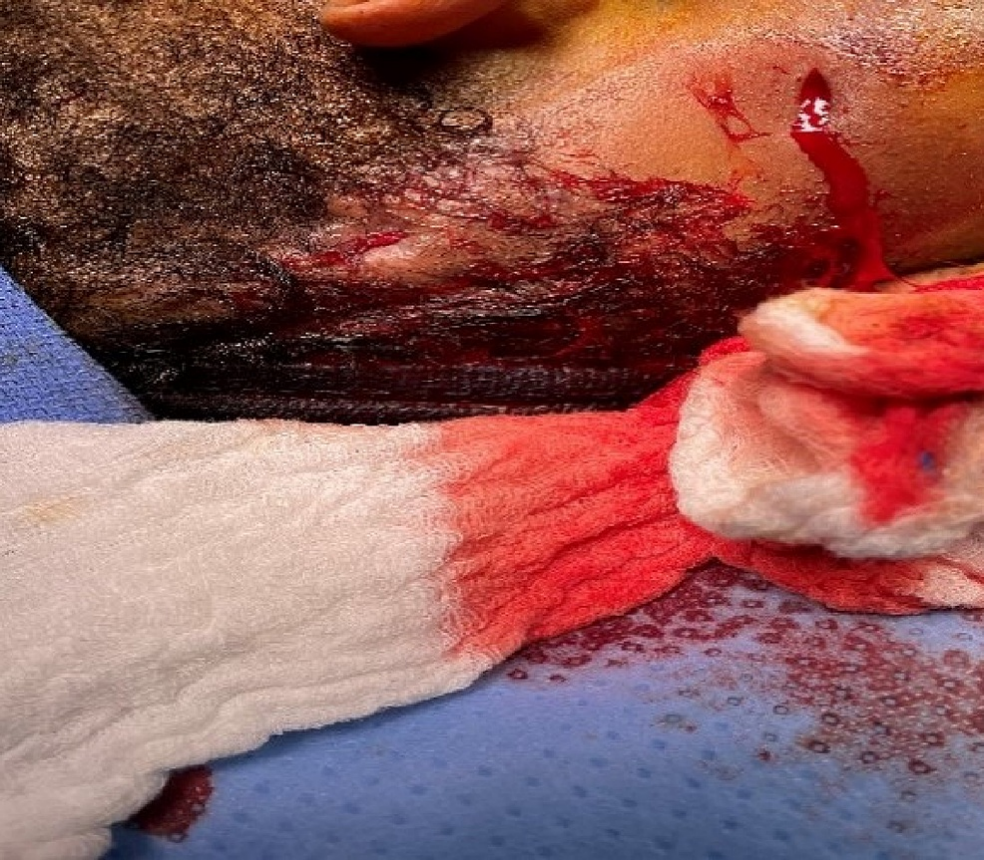
HS lesion status post incision and drainage. HS: hidradenitis suppurativa

ENT was consulted and incision and drainage (I&D) of the deep neck abscess was performed (Figures 5, 6). No growth was noted from culture. Antibiotic regimen upon discharge included ciprofloxacin HCL 500 mg (1 tablet orally twice daily), clindamycin 300 mg (1 capsule orally three times daily), and linezolid 600 mg (1 tablet orally twice daily) to be taken for 14 days post-discharge.

**Figure 7:**
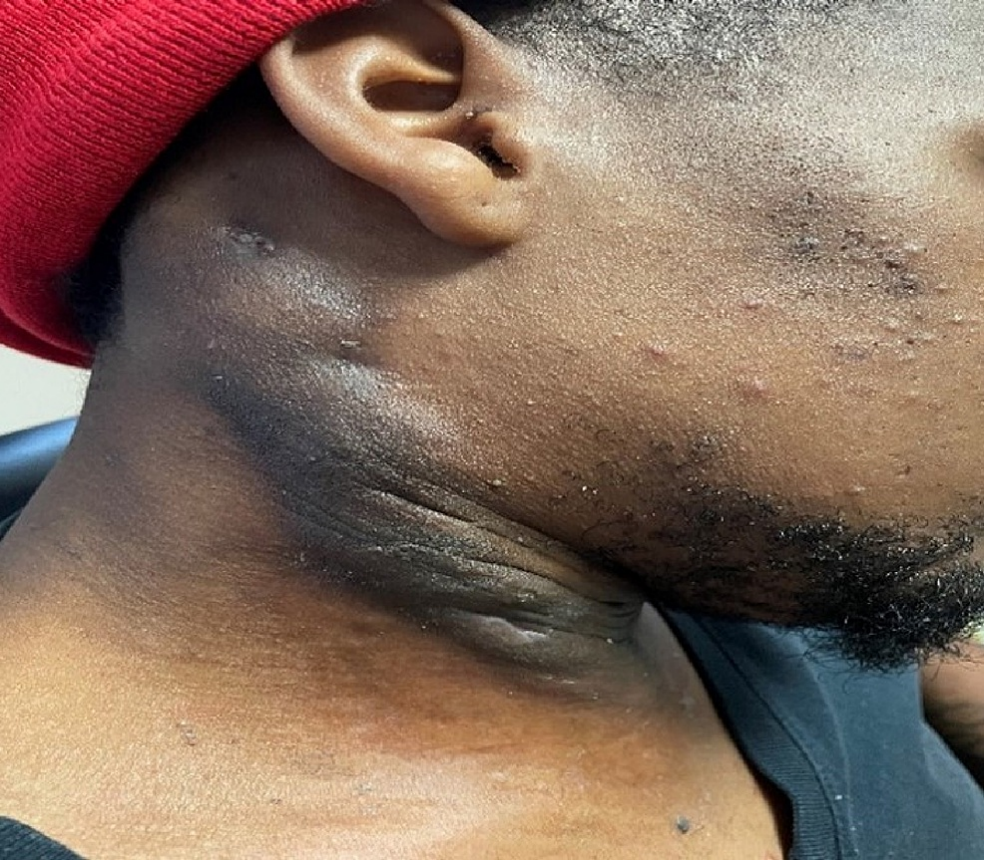
HS lesion at one-week post-operative follow-up. HS: hidradenitis suppurativa

**Figure 8:**
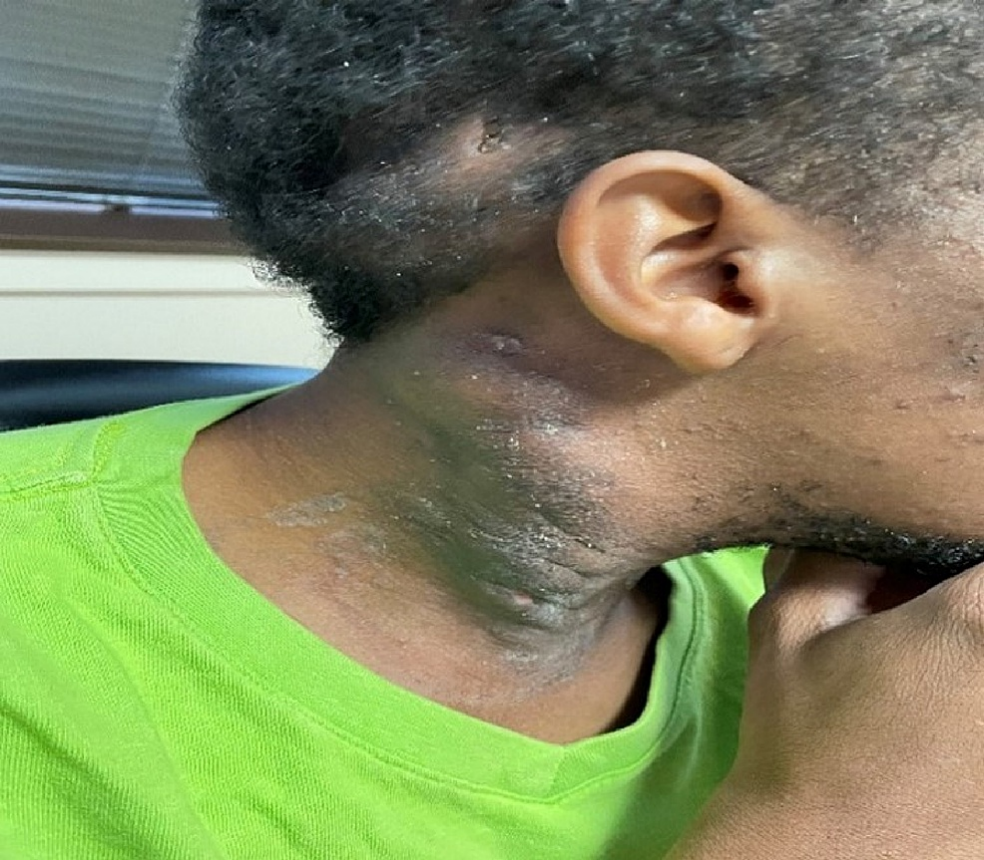
HS lesion at three weeks post-operative with resolution of drainage and swelling. HS: hidradenitis suppurativa

**Figure 9:**
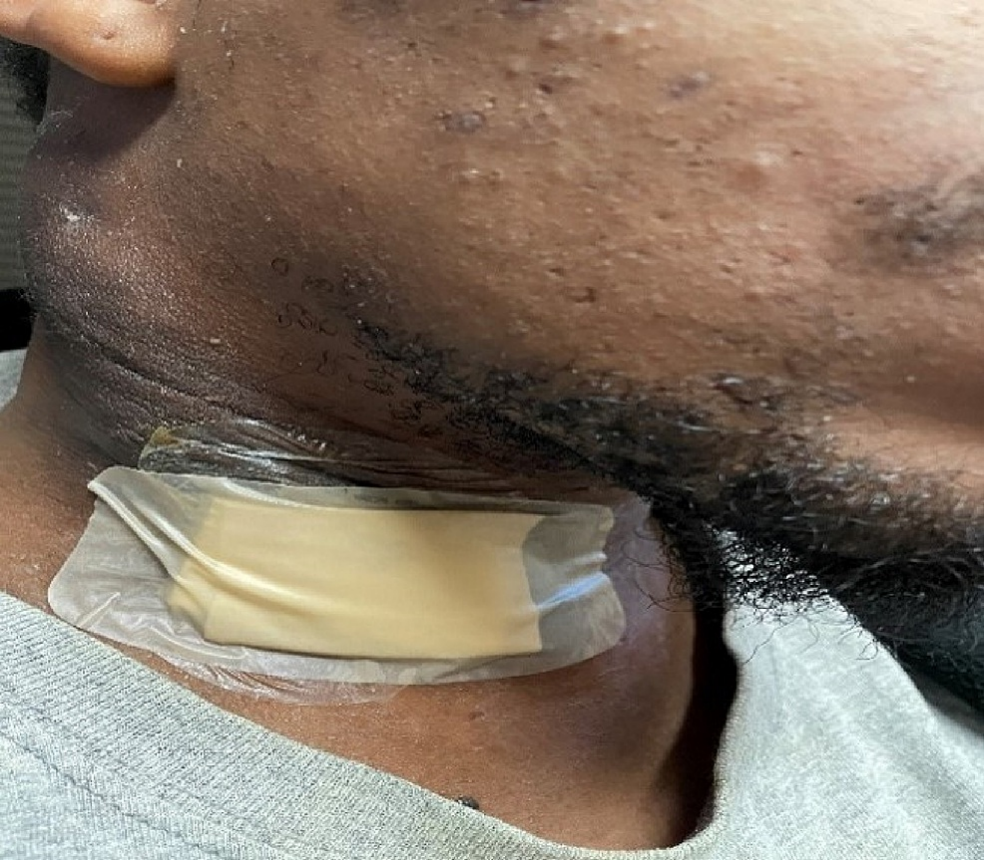
Patient at one month post-operative with dry dressing and no drainage.

The patient continued to follow up with ENT for wound care and showed gradual improvement with drainage and pain (Figures 7-9). The pain was managed with Tylenol #3 as needed and the antibiotic regimen remained the same.

**Figure 10:**
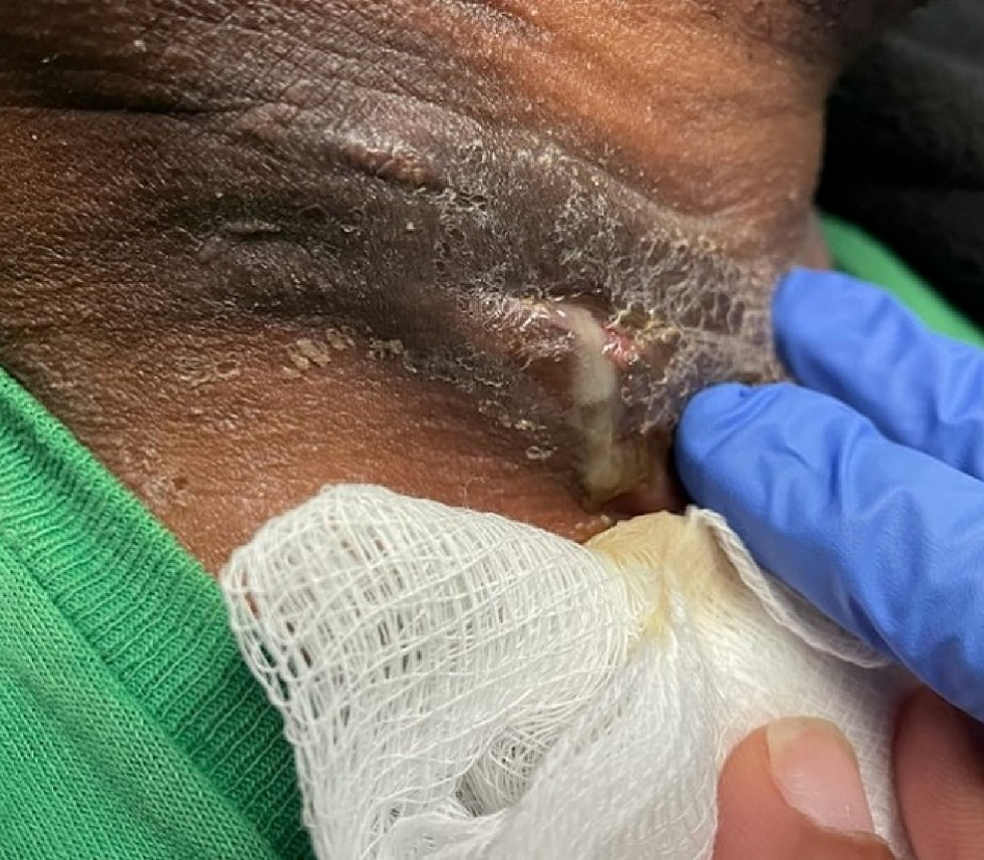
Hs lesion at six weeks post-operative with drainage from incision site. HS: hidradenitis suppurativa

Six weeks post-operative, the patient presented to the clinic with drainage from the incision site that eventually resolved with a six-week period of antibiotics, ciprofloxacin HCL 500 mg 1 tablet orally twice daily, and proper wound care (Figure 10). 

## Discussion

Hidradenitis suppurativa (HS) is a chronic inflammatory disease that is characterized by recurrent painful nodules, draining sinus tracts, and abscesses [1]. The disease has an estimated global prevalence rate of up to 4% and primarily affects the axillary, perineal, inguinal, intermammary, and inframammary regions [2]. This specific topography mirrors the anatomical distribution of apocrine sweat glands [4]. This pattern of distribution is the reason why HS was once thought to be an infectious process of the apocrine glands. However, pathologists have demonstrated that the primary histological event occurs in the follicular duct [4]. This shared similarity in the pathogenic process with acne gave rise to the name “acne inversa.” The dismissal of apocrine sweat glands as the driving force in the pathogenesis of HS is mainly because apocrine sweat glands are spared in the initial inflammatory and destructive process [4]. Pathogenesis is initiated rather by follicular hyperkeratosis with plugging and dilatation of the hair follicle. This leads to inflammation, abscess, and sinus tract formation. The involvement of the apocrine glands is a result of granulomatous inflammation in the deep structures of the skin.

While the etiology of hidradenitis suppurativa is still unknown, there are factors that increase the chance of developing the disease. HS is more likely to develop in women than in men and most commonly occurs between the ages of 18 years and 29 years. That said, HS can occur at any age but onset at an early age increases the risk of developing the widespread disease. Obesity has also been identified as a risk factor for the disease. Several studies have shown a relationship between hidradenitis suppurativa and being overweight [5]. Other risk factors include smoking and having a family history.

The severity of HS is rated using the Hurley staging system. There are three Hurley stages. Hurley stage I is characterized by a single nodule with no sinus tracts, stage II has more than one nodule with little tunneling, and stage III has multiple nodules with a lot of sinus tracts and scars involving an entire area of the body [6]. Phenotypic variation occurs in HS, which may make severity assessment difficult. The high phenotypic variation in HS is likely due to multiple underlying etiologies, given that this is still poorly understood. In this case, the patient presented with a large neck abscess extending from the right mastoid to the anterior neck, multiple lesions, and extensive sinus tract formation indicative of Hurley stage III. 

**Figure 11:**
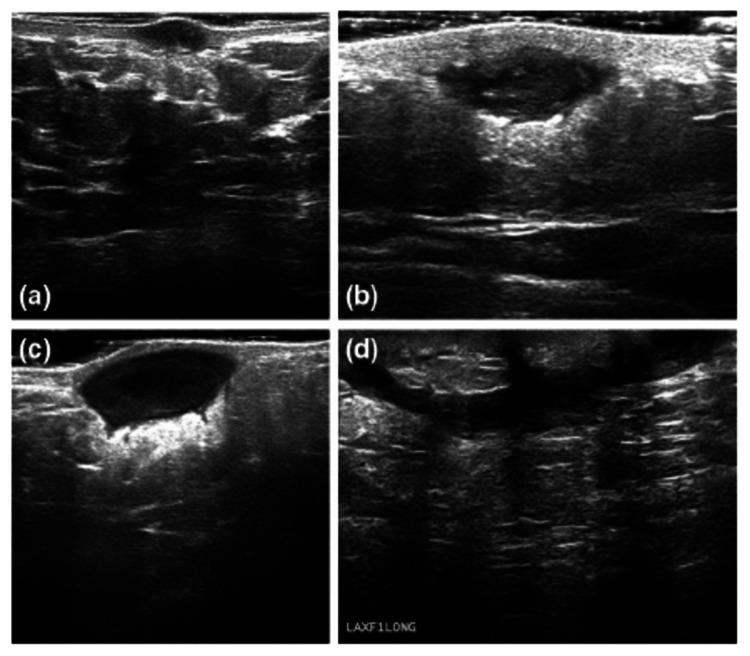
Ultrasound showing the types of HS lesions - (a) superficial nodule, (b) deep nodule, (c) abscess, and (d) tunneling. HS: hidradenitis suppurativa

The diagnosis of hidradenitis suppurativa is made clinically and is based on three important clinical features; typical lesions, location, and chronicity [3]. However, diagnosing HS can be challenging. A diagnostic delay is evident partly due to early lesions of HS mimicking other skin conditions. Hidradenitis suppurativa is often misdiagnosed as boils or furunculosis on initial presentation [6]. The consequence of which is prolonged patient suffering. The average global diagnostic delay of hidradenitis suppurativa is seven to 10 years leading to increased efforts to develop effective screening methods [6]. CT scans and ultrasounds are emerging as important diagnostic tools, especially in the case of deep-seated lesions (Figure 11). Imaging can be used to define lesion morphology and depth. It can be used to show fluid collections that are not evident, increased dermal thickness and follicular dilation seen in the early stages of the disease. This could aid with diagnostic delay and increase the precision of treatment.

Multiple comorbidities are associated with hidradenitis suppurativa, and persistent HS often results in complications. Obesity, metabolic syndrome, inflammatory bowel disease, and spondyloarthropathy are most noteworthy. Complications include infection, scarring with associated restricted limb mobility, obstructed lymph drainage, fistulas, and social isolation. The size and location of the abscess in this case led to the development of dysphagia; a complication not normally seen in hidradenitis suppurativa. The area affected by hidradenitis suppurativa has increased susceptibility to infection. As HS wounds heal scars and skin changes are left behind which can result in restricted and painful movement. Scarring of tissues can also interfere with lymph drainage, as the most common sites that HS affects also contain many lymph nodes. Interference with lymph drainage results in swelling in the arms, legs, or genitals. The psychosocial effect of HS is devastating because of the associated pain, malodorous discharge, and scarring [6]. The location of lesions coupled with drainage and odor can result in the reluctance to visit public spaces and the onset of depression.

Hidradenitis suppurativa is not curable, but effective treatments to control disease and improve symptoms are available. Treatment is based on disease severity according to Hurley staging. For mild disease, lifestyle changes including weight loss, smoking cessation, a healthy diet, and reducing mechanical stress on the skin by wearing loose-fitting clothing may be helpful. Although not a primarily infectious disease, HS is improved with antibiotics. Mild symptoms of HS can be managed with a topical antibiotic, but for the more widespread disease, oral antibiotics, such as doxycycline, clindamycin, rifampin or a combination of both is necessary (Table 1) [5]. Patients with severe disease may need to take antibiotics for months for symptom relief. Interestingly, drugs that are conducive to infection such as immunosuppressives, anti-tumor necrosis factor (anti-TNF) agents, and corticosteroids may improve the disease. Surgical intervention allows for the excision of isolated lesions. There is no consensus on the most appropriate time for surgery, but the current approach is to utilize surgery in patients who fail to respond to multiple medical therapies. These patients tend to be those in Hurley stage III as is the case with this patient. HS has a high rate of recurrence with medical therapies as well as surgical intervention. Following surgical incision and drainage, the patient in this case required prolonged antibiotic therapy for resolution of symptoms.

**Table 1:** Recommendations for systemic antibiotic use in HS HS: hidradenitis suppurativa

Systemic Antibiotics	Recommendation
Tetracyclines	Mild to moderate HS, up to 12 weeks duration
Clindamycin and rifampin combination	2nd line treatment in mild to moderate HS
Moxifloxacin, metronidazole and rifampin	2nd line or 3rd line treatment in moderate to severe HS
Dapsone	May be used for long term maintenance therapy

Wide surgical excision is indicated in severe chronic HS and is associated with lower rates of recurrence. However, the sites where this surgical technique is commonly used include the axillary, perineal, and gluteal regions. Wide excision requires extensive reconstruction and the use of skin grafts for closure of the resulting defect. This is a case report of HS in the neck region which is a rare location. The widespread involvement of the left posterior scalp, right posterior, and anterior neck, down to the level of the sternoclavicular joint makes wide excision an unlikely option. The recurrent nature of HS as well as the lack of curative therapies makes the treatment of the disease challenging [6].

## Conclusions

The diagnosis of HS is made clinically and is based on typical lesions, location, and chronicity. However, phenotypic variation makes diagnosis and severity assessment difficult. Furthermore, a diagnostic delay is evident partly due to early lesions of HS mimicking other skin conditions. In modern medicine, CT scans and ultrasounds are emerging as important diagnostic tools, especially in the case of deep-seated lesions. Multiple comorbidities are associated with HS, most notably obesity, metabolic syndrome, and other risk factors for cardiovascular disease. Persistent hidradenitis suppurativa often results in complications, including infection, scarring, obstructed lymph drainage as well as social isolation leading to a poor mental status. The recurrent nature of HS as well as the lack of curative therapies makes the treatment of the disease challenging.
